# Pre-surgery dietician counseling can prevent post-thyroidectomy body weight gain: results of an intervention trial

**DOI:** 10.1007/s12020-023-03365-z

**Published:** 2023-04-19

**Authors:** Laura Croce, Cristina Pallavicini, Noemi Busca, Benedetto Calì, Giuseppe Bellastella, Francesca Coperchini, Flavia Magri, Luca Chiovato, Hellas Cena, Mario Rotondi

**Affiliations:** 1 Department of Internal Medicine and Therapeutics, University of Pavia, Pavia, 27100 Italy; 2 Istituti Clinici Scientifici Maugeri IRCCS, Unit of Endocrinology and Metabolism, Laboratory for Endocrine Disruptors, Pavia, 27100 Italy; 3 NBFC, National Biodiversity Future Center, Palermo, 90133 Italy; 4 Istituti Clinici Scientifici Maugeri IRCCS, Department of General and Minimally Invasive Surgery, Pavia, 27100 Italy; 5 Department of Advanced Medical and Surgical Sciences, University of Campania “L. Vanvitelli”, Naples, Italy; 6 Laboratory of Dietetics and Clinical Nutrition, Department of Public Health, Experimental and Forensic Medicine, University of Pavia, 27100 Pavia, Italy; 7 Istituti Clinici Scientifici Maugeri IRCCS, Clinical Nutrition and Dietetics Service, Unit of Endocrinology, 27100 Pavia, Italy

**Keywords:** Body weight gain, Thyroidectomy, Diet Counseling, Obesity, BMI

## Abstract

**Purpose:**

It is widely accepted that patients experience weight gain after total thyroidectomy, and preventive measures should be recommended.

**Methods:**

A prospective study was designed to assess the efficacy of a dietetic intervention to prevent post-thyroidectomy weight gain in patients undergoing surgery for both benign and malignant thyroid conditions. Patients undergoing total thyroidectomy were prospectively and randomly assigned to receive a personalized pre-surgery diet counseling (GROUP A) or no intervention (GROUP B), according to a 1:2 ratio. All patients underwent follow-up with body-weight measurement, thyroid function evaluation and lifestyle and eating habits assessment at baseline (T0), 45 days (T1) and 12 months (T2) post-surgery.

**Results:**

The final study group encompassed 30 patients in Group A and 58 patients in Group B. The two groups were similar in terms of age, sex, pre-surgery BMI, thyroid function and underlying thyroid condition. The evaluation of body weight variations showed that patients in Group A did not experience significant body weight changes at either T1 (*p* = 0.127) nor T2 (*p* = 0.890). At difference, patients in Group B underwent a significant body weight increase from T0 to both T1 (*p* = 0.009) and T2 (*p* = 0.009). TSH levels were similar in the two groups, both at T1 and T2. Lifestyle and eating habits questionnaires failed to register any significant difference between the two groups, apart from an increase in sweetened beverages consumption in Group B.

**Conclusions:**

A dietician counseling is effective in preventing the post-thyroidectomy weight gain. Further studies in larger series of patients with a longer follow-up appear worthwhile.

## Introduction

An increase in body weight is experienced by the majority of patients undergoing total thyroidectomy [1]. Although this gain of weight is usually modest in entity, it may acquire clinical relevance, especially in populations at high risk for overweight and obesity [2, 3]. The mechanisms underlaying the post-thyroidectomy gain of weight are not completely understood. Early studies investigated Graves’ disease patients [4, 5] and concluded that a regain of body weight lost during the hyperthyroid phase of the disease was, at least in part, responsible of this phenomenon. Subsequent studies reported significant weight gains also in patients undergoing thyroidectomy for multinodular non-toxic goiter [6, 7] and thyroid cancer [8–12], and even in patients addressed to post-thyroidectomy TSH suppressive LT-4 therapy. In a previous study [13] we retrospectively evaluated body weight changes in patients undergoing thyroidectomy for a wide spectrum of thyroid diseases. We found a significant weight gain after thyroidectomy, which was not dependent on the underlying thyroid disease and/or post-surgery TSH levels. Short-term body weight change (i.e. 40–60 days after thyroidectomy) was the only predictive factor for the patients’ weight gain after one year. Since then several studies [14] and meta-analysis [1, 11] confirmed a modest but significant increase in body weight occurring post-thyroidectomy. Moving from this observation, preventive measures should be implemented. To this aim we designed a prospective randomized study to assess whether a dietician counseling might prevent the post-thyroidectomy body weight gain.

## Materials and methods

Patients were enrolled among those undergoing thyroidectomy at the Surgery Unit of the Istituti Clinici Scientifici Maugeri, I.R.C.C.S. (Pavia, Italy), between February the 1^st^ and the June the 30^th^ in 2021. All patients were Caucasian, lived in Pavia or the surrounding Northern Italy area. Inclusion criteria were: availability of 1) anthropometric data (such as sex, age, body mass index, BMI), 2) data regarding lifestyle (physical activity and smoking habit as assessed during the pre-surgical (two weeks before) visit; 3) pre-surgery thyroid function parameters and positivity for anti-thyroglobulin (Tg Ab) and/or anti-thyroperoxidase (TPO Ab) antibodies. Total thyroidectomy was performed in all patients.

Weight was measured on two different weight scales, which are periodically checked for their accuracy by an agency of standards, asking the participant to stand still in the center of the scale platform with light clothing with 10 cm gap between the heels, making sure the weight was equally distributed on both legs. Height was measured by means of a stadiometer on the individuals standing up tall, keeping their heels on the ground flat against the tool, and barefoot, to the nearest 0.1 cm [15]. BMI was then calculated as weight in kilograms divided by the height divided by height in meters squared.

Enrolled patients were randomly assigned to receive a personalized pre-surgery diet counseling (GROUP A) or no intervention (GROUP B), according to a 1:2 ratio. All patients underwent endocrinological follow-up at baseline (within a week from surgery), 45 days and 12 months post-surgery in the Endocrinology Outpatient Clinic. Exclusion criteria during follow-up included: pregnancy, inter-current illnesses influencing body weight, and bariatric surgery.

All subjects gave their informed consent to participate in the study, which was performed in accordance with the guidelines of the Declaration of Helsinki. This study was formally approved by the Istituti Clinici Scientifici Maugeri IRCCS Ethical Committee (Protocol number CE 737).

## Dietic intervention

All the patients (Group A) received an individual nutritional counseling, performed by a registered clinical dietitian, within one week pre-surgery (T0). The dietitians analyzed the patient’s dietary pattern and habits, estimating intake frequencies of all the food groups throughout a validated questionnaire, the “QueMD MODV6” [16] in order to investigate the main mistakes and wrong behaviors. For each patient we calculated a score ranging from 0 (minimal adherence to Mediterranean diet) to 9 (maximal adherence). During the counseling, the dietitians provided advice for better food choices according to the Italian National Dietary Guidelines. Each patient was offered a “Healthy and Correct nutrition” leaflet, that was explained by the dietitians, providing also detailed information regarding the risk of weight gain post-thyroidectomy and advising each patient to monitor the body weight regularly on the same weight scale, at the same time and week day once a week. Moreover, the dietitians also administered a custom-made questionnaire regarding lifestyle changes after thyroidectomy, including a question on physical activity and a question on smoking habits.

Both Groups, A and B, attended 40–60 days post-thyroidectomy (T1) and 1 year after (T2) dietetic consultations, which included evaluation of body weight, BMI, level of physical activity, smoking habitude and dietary modification by the QueMD MODV6 questionnaire.

## Endocrine follow-up

All patients attended regular endocrinological follow-up at T1 and T2, according to standard clinical practices. After surgery all patients were treated with levothyroxine at replacement or mildly TSH-suppressive doses according to a benign or malignant histology. Thyroid function parameters were measured at 40–60 days and at 12 months after surgery in all patients. Patients who, on the basis of the 40–60 days post-surgery TSH levels, required levothyroxine dose adjustments were further tested for thyroid function parameters two months later.

### Statistical analysis

Statistical analysis was performed using the SPSS software (SPSS, Inc., Evanston, IL). Between groups comparisons were performed by Student’s *t* test for unpaired data or by Mann–Whitney *U*-test according to a normal or a nonparametric distribution of the tested variable. Frequencies among groups were compared by χ^2^ test with Fisher’s correction, when appropriate. A *p*-value < 0.05 was considered statistically significant.

## Results

Ninety patients (30 in Group A and 60 in Group B) fulfilled the inclusion criteria. Two patients in Group B were excluded during the follow-up: one to the occurrence of pregnancy, and another due to treatment with metformin a potentially body-weight modifying drug. The final study group encompassed 88 patients (30 in Group A and 58 in Group B). Table 1 shows the pre-surgery anthropometric and clinical data of patients. Patients in the two groups were similar in terms of sex, age, underlying thyroid disease, pre-surgery weight and BMI, pre-surgery TSH and pre-surgery positive tests for Tg Ab or TPO Ab.

**Table 1:** Anthropometric and clinical data of patients in Group A and B

	Group A	Group B	*p* value
Number of patients	30	58	
Sex (Male/Female)	10/20	12/46	0.194
Age (years, mean ± SD)	51.3 ± 12.4	52.0 ± 11.7	0.699
Pre-surgery body weight (kg) (mean ± SD)	71.5 ± 14.1	67.8 ± 15.5	0.275
Pre-surgery BMI (kg/m^2^) (mean ± SD)	27.1 ± 4.4	26.2 ± 5.7	0.275
Pre-surgery TSH (µU/mL) [median (IQR)]	1.32 (0.63–2.50)	0.74 (0.42–1.86)	0.118
Pre-surgery TgAb/TPOAb positivity *N* (%)	9 (30.0%)	16 (27.6%)	0.812
Thyroid disease *N* (%)			
Non-toxic nodular goiter	17 (56.7%)	32 (55.2%)	0.999
Toxic nodular goiter	2 (6.7%)	4 (6.9%)	
Differentiated thyroid cancer	7 (23.3%)	14 (24.1%)	
Graves’ disease	4 (13.3%)	8 (13.8%)	

*BMI* body mass index, *IQR* interquartile range, *SD* standard deviation, *TSH* thyroid stimulating hormone, *TgAb* anti-thyroglobulin antibody, *TPOAb* anti-thyroperoxidase antibody

### Body weight changes

We compared body weight changes from pre-surgery (T0) to T1 and T2 between the two groups. Patients in Group A did not experience significant body weight changes at either T1 or T2. In particular, mean body weight was 71.5 ± 14.1 kg at T0, 71.9 ± 14.2 kg at T1 (*p* = 0.127) and 71.4 ± 13.9 at T2 (*p* = 0.890). At difference, patients in Group B underwent a significant body weight increase from T0 to both T1 and T2. In particular, mean body weight was 67.8 ± 15.5 kg at T0, 68.8 ± 15.5 kg (*p* = 0.009) at T1 and 69.3 ± 15.8 kg (*p* = 0.009) at T2. Of note, the serum levels of TSH were similar in the two groups, both at T1 (median TSH = 2.30 µU/ml (IQR 0.38–5.87) in Group A vs median TSH = 2.50 µU/ml (IQR 0.72–5.47) in Group B (*p* = 0.646) and at T2 (median TSH = 1.86 µU/ml (IQR 0.25–4.11) in Group A vs a median TSH = 1.40 µU/ml (IQR 0.15–2.95) in Group B (*p* = 0.469).

**Fig. 1:**
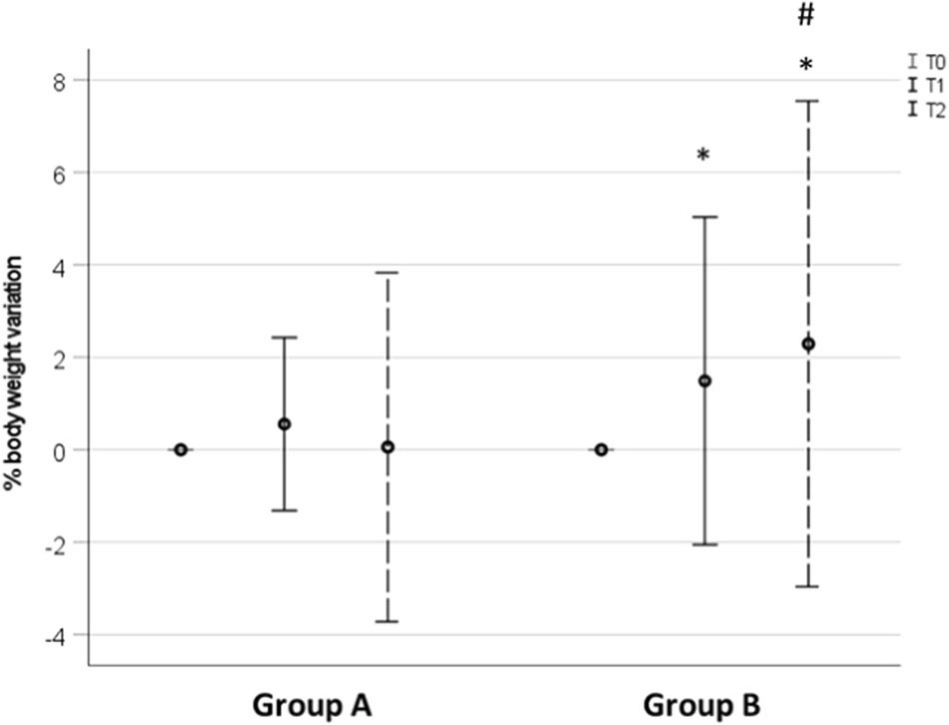
Percentage body weight variation between T0 and T1 and between T0 and T2 in the two study groups. Data are represented as mean ± standard deviation. *p < 0.05 vs T0; #*p* < 0.05 vs Group A at T2

As shown in Fig. 1, body weight changes expressed as mean ± SD were: 0.55 ± 1.87% between T0 and T1 and 0.06 ± 3.77% between T0 and T2 in Group A; 1.49 ± 3.55% between T0 and T1 and 2.29 ± 5.25% between T0 and T2 in Group B.

**Fig. 2:**
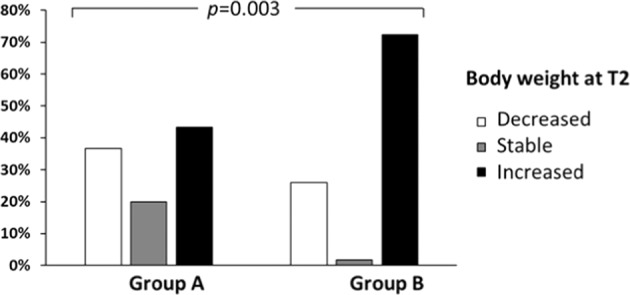
Histogram representing the percentage of patients experiencing decreased, stable or increased body weight at T2 when compared to T0 in Group A and Group B

As shown in Fig. 2, we compared the rate of patients experiencing a decreased, increased or a stable body weight at T2 in Group A vs. Group B. We found a significant different distribution (*p* = 0.003). In particular, a higher percentage of patients experiencing body weight increase was observed in Group B (72.4%) when compared with Group A (43.3%); whereas 36.7% in Group A versus 25.9% in Group B experienced a reduction in body weight. Stable body weight was recorded in 20.0% of patients in Group A versus 1.7% in Group B.

### Lifestyle, dietary pattern and habits changes

The analysis of lifestyle and QueMD MODV6 questionnaires throughout the study span failed to register any significant difference between the two groups, both at baseline (mean score 6.90 ± 1.32 in Group A vs 7.07 ± 1.25 in Group B, *p* = 0.558), at T1 (6.90 ± 1.27 in Group A vs 7.05 ± 1.29 in Group B, *p* = 0.660) and at T2 (6.93 ± 1.36 vs 7.02 ± 1.44, *p* = 0.793). Moreover, no significant variations in time was observed in both groups between T0 and T2 (for Group A, *p* = 0.994; for Group B: *p* = 0.616) Lifestyle assessment indicated that patients’ attitude towards physical activity and/or smoking habits were similar between the two groups at baseline and during follow-up. However, 3 patients (1 in Group A and 2 in Group B) out of 29 active smokers (10 in Group A and 19 in Group B) quit smoking after surgery and did not relapse throughout the follow-up (*p* = 0.964). Among the 22 patients used to weekly physical activity (>2 h/week) pre-surgery, 20 patients (90.9%) stopped physical activity for nearly 1-2 months post-surgery. Among these, 6 patients belonged to Group A and 14 to Group B) (*p* = 0.660). Furthermore, 5 out of those 20 patients (25.0%, 2 in Group A and 3 in Group B, *p* = 0.573) did not resume the previous level of physical activity during follow-up. For food frequencies, the only difference was registered for sweetened beverages consumption (mainly fruit juices). In details, among the 19 usual consumers of sweetened beverages (7 in Group A and 12 in Group B), an increase of daily intake was reported in 2 out of 7 (28.6%) and 9 out of 12 (75.0%) in Group A and Group B, respectively (*p* = 0.048). Among the patients who were not previously habitual consumers of sweetened beverages, in Group A and Group B, only 1 patient out of 23 (4.3%) and 4 out of 45 patients (8.8%) started consumption of sweetened beverages after surgery, respectively (*p* = 0.497).

## Discussion

The results of the present prospective, randomized study demonstrate that a pre-surgery dietician intervention may effectively prevent the post-thyroidectomy weight gain. In spite of this statistically significant result, questionnaires regarding diet habits and lifestyle failed to show any significant difference between intervention and control group. A trend towards greater consumption of sweetened beverages and diminished physical activity was observed in the non-intervention group, although statistical significance was not reached, probably due to the small sample size. Other potential confounders (i.e. age, sex, underlying thyroid pathology and baseline BMI) were similar between the two groups.

Among previous studies investigating the causes of post-thyroidectomy weight gain, some highlighted the role of post-surgery hypothyroidism [6, 11], and other showed a lower body weight gain in patients receiving TSH-suppressive therapy for thyroid cancer [10]. In line with another group of previous studies [8, 12, 13], in the present prospective randomized trial we found no relationship between post-surgery serum levels of TSH and body weight changes. It is interesting noting that, at any time point, similar serum levels of TSH were found during follow-up in the two groups of patients.

Overall life-style and dietary pattern assessment failed to register significant differences between the two groups and between pre-surgery and post-surgery. This could be due to the limited sample size, however it is undeniable that some of those factors, including failure in maintaining the same physical activity levels as before surgery, increased intake of comfort food as sweeten beverages and, last but not least, quitting smoking, might contribute to the thyroidectomy-related increase in body weight. Several studies reported increased complain for post-surgery asthenia in patients performing thyroidectomy [17–20]. This appears not of negligible relevance, in that, it could, at least in part, explain the here reported trend towards a reduction in physical activity in these patients [21]. As for the increase in consumption of sweetened beverages, patients referred that, in the early post-surgery period (i.e. starting from the in-patient stay), drinking cold and sweet beverages was associated with a relief in neck discomfort probably due to post-surgical inflammation.

In the present study, the mean body weight increase in the non-intervention group was +1.5 kg at 12 months after surgery, which is in line with what reported by a recent meta-analysis [1]. Although the entity of this post-thyroidectomy weight gain might appear small, it should not be considered clinically irrelevant. Indeed, epidemiologic studies suggest that, in the general population, every 1 kg increase in body weight is associated with a nearly 4.5% increase in the prevalence of Type 2 diabetes [22–24]. Moreover, similar to other countries, the prevalence of obesity in the Italian population is high; thus, preventing any avoidable weight gain appears worthwhile.

A further aspect to be considered is the notion that body weight gain is among the main complaints of patients addressed to thyroidectomy, potentially contributing to a reduced quality of life according to some studies [25, 26]. Thus, the observation that a pre-surgery dietician counseling can prevent the post-thyroidectomy increase in body weight is clinically relevant.

The main limitation of the present study stems from the rather low number of enrolled patients, which could induce to consider the results as rather preliminary. However, the effectiveness of dietician counseling in patients undergoing thyroidectomy appears worth to be reported.

In conclusion, the results of our study suggest that a dietician counseling is effective in preventing the post-thyroidectomy body weight gain. Further studies in larger series of patients and with a longer follow-up appear worthwhile.

## Data Availability

Some or all data used during the study are available from the corresponding author by request.
